# A Review of Perovskite Photovoltaic Materials’ Synthesis and Applications via Chemical Vapor Deposition Method

**DOI:** 10.3390/ma12203304

**Published:** 2019-10-11

**Authors:** Xia Liu, Lianzhen Cao, Zhen Guo, Yingde Li, Weibo Gao, Lianqun Zhou

**Affiliations:** 1 Department of Physics and Optoelectronic Engineering, Weifang University, Weifang 261061, Shandong, China; liuxia@wfu.edu.cn (X.L.); wfulyd@163.com (Y.L.); 2 Chinese Academy of Sciences Key Lab of Bio-Medical Diagnostics, Suzhou Institute of Biomedical Engineering and Technology, Chinese Academy of Sciences, Suzhou 215163, Jiangsu, China; 3 Division of Physics and Applied Physics School of Physical and Mathematical Sciences Nanyang Technological University, Singapore 637371, Singapore; 4 Shandong Guo Ke Medical Technology Development Co., Ltd., Jinan 250001, Shandong, China; 5 Zhongke Mass Spectrometry (Tianjin) Medical Technology Co., Ltd., Tianjin 300399, China

**Keywords:** atmospheric pressure CVD, low pressure CVD, hybrid CVD, aerosol assisted CVD, pulsed CVD, perovskite photovoltaic nanomaterials, stabilization, structural design, performance optimization, solar cells

## Abstract

Perovskite photovoltaic materials (PPMs) have emerged as one of superstar object for applications in photovoltaics due to their excellent properties—such as band-gap tunability, high carrier mobility, high optical gain, astrong nonlinear response—as well as simplicity of their integration with other types of optical and electronic structures. Meanwhile, PPMS and their constructed devices still present many challenges, such as stability, repeatability, and large area fabrication methods and so on. The key issue is: how can PPMs be prepared using an effective way which most of the readers care about. Chemical vapor deposition (CVD) technology with high efficiency, controllability, and repeatability has been regarded as a cost-effective road for fabricating high quality perovskites. This paper provides an overview of the recent progress in the synthesis and application of various PPMs via the CVD method. We mainly summarize the influence of different CVD technologies and important experimental parameters (temperature, pressure, growth environment, etc.) on the stabilization, structural design, and performance optimization of PPMS and devices. Furthermore, current challenges in the synthesis and application of PPMS using the CVD method are highlighted with suggested areas for future research.

## 1. Introduction

**Figure 1:**
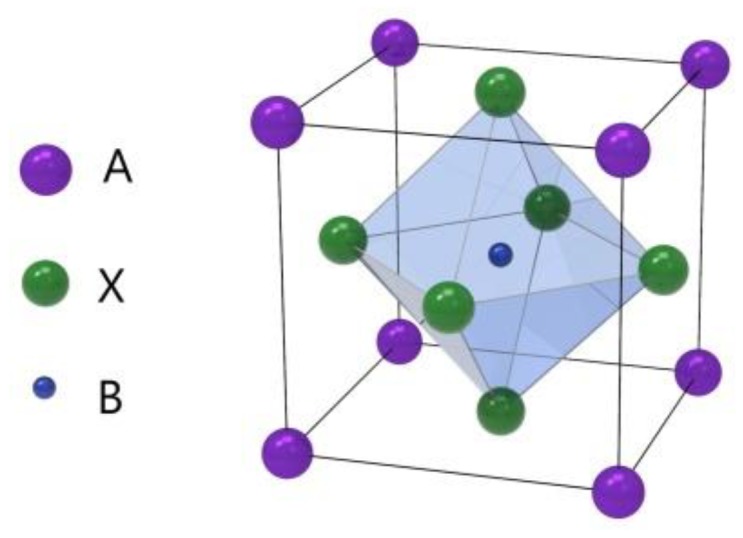
Crystal structure of the perovskite ABX_3_ form.

Perovskite is a kind of material with the same crystal structure as calcium titanate (CaTiO_3_), which was discovered by Gustav Rose in 1839 [1]. The perovskite material (PM) structure formula is generally ABX_3_, where A and B are two cations and X is anion, as shown in Figure 1 [2]. This unique crystal structure gives it many unique physical and chemical properties, such as broadband bandgap tunability, high carrier mobility, high optical gain, strong nonlinear response, and simplicity of their integration with other types of optical and electronic structures [3,4,5,6,7,8]. Due to its excellent physical and chemical properties, especially optical properties, perovskite materials are now widely applied for constructing photovoltaic solar cells (PSCs) [2,9,10,11].

Up to now, great progress has been made in the preparation and application of bulk PPMs. The perovskite family now includes hundreds of substances, ranging from organic materials, inorganic materials to organic–inorganic materials, from polycrystalline film to bulk single crystal [12]. However, introduction of many defects and grain boundaries in three-dimensional (3D) bulk PPMS is unavoidable [13], which inevitably degrade its optoelectronic properties. Recently, low-dimensional PPMS with high quality have begun to receive increasing attention, primarily for their tunable optical and electronic properties due to quantum-size effects as well as their enhanced photoelectric performance compared with bulk materials. Moreover, perovskites are considered to be a class of important low-dimensional layered materials, whose optical properties can be controlled by varying the number of layers, particularly at thicknesses lower than their exciton Bohr radius. Therefore, the shape-, size-, and thickness-controlled synthesis of low-dimensional PPMs has recently been a hot topic of research. Various low-dimensional morphologies, including zero-dimensional (0D) morphologies, such as quantum dots and nanoparticles; one-dimensional (1D) morphologies, such as nanowires and nanorods; and two-dimensional (2D) morphologies, such as nanoplates and nanosheets, have been prepared and applied [14,15,16,17,18,19,20,21].

Due to potential applications of perovskite materials (PMs) in photovoltaics wide spread interest has focused on exploring various synthesis methods to obtain high quality perovskite materials for constructing designed devices with improved performance. Up to now, many synthesis methods—such as solution process [4,22,23,24,25,26], thermal evaporation process [27], flash evaporation [28], doctor-blade coating method [29], slot-die coating method [30], spray deposition [31], ink-jet printing method [32], vapor-assisted solution process (VASP) [33], and so on—have been developed for preparing PPMs with well-designed dimensions for exploring factors which plays key role in dominating their properties. Among various approaches, the most studied solution method not only achieves highly efficient perovskite solar cells (PSCs) but also has a cost advantage. However, the uncontrolled rapid liquid reaction often generates rough, porous, and less-stable perovskite films with incomplete conversions, resulting in large variations on film morphology and Photovoltaic (PV) response [24,25,26]. Inversely, thermal evaporation can generate high-quality perovskite films with smooth and pinhole-free morphologies. However, there are a few of disadvantages in thermal evaporation process, such as low material utilization, and high equipment investment and energy consumption, hampering its further application [27,28]. The rest technologies often produce poor perovskite film quality, and are also difficult to scale up [29,30,31,32]. VASP methods require manipulations protecting atmosphere, and show poor controllability and versatility, unsuitable for their mass production [33]. Therefore, development of a new class of advanced fabrication technology shall be critical for the future commercialization of PPMs.

Soon after, as an evolution of VASP, an array of CVD techniques [2,34,35,36,37,38,39]—such as one-step and two-step tubular CVD, in situ tubular CVD (ITCVD), aerosol-assisted CVD (AACVD), hybrid physical-chemical vapor deposition (HPCVD), plasma-enhanced CVD (PECVD), and so on—were developed to grow high-quality PPMs. At present, CVD of perovskites is a promising alternative to solution based methods of fabrication due to the relative ease of patterning, the ability to batch process, the wide range of material compatibility, and the potential for uniform large-area deposition.

Here, in this review paper, CVD method—as one of the key technologies for preparing many kinds of advanced semiconductor materials with excellent properties—is mainly mentioned for preparing PPMs to construct optimal devices. For details, in this topical review, we present the recent developments of synthesis and application of various PPMs via the CVD method. We begin with the characteristics, classification, reaction process, and application of CVD method. In the following section, we mainly summarize the influence of different CVD technologies and important experimental parameters (temperature, pressure, growth environment, etc.) on the PPMs and devices. In particular, we aim to show why CVD technology can improve the stability of materials and devices, and can help material and device design, thus improving material and device performance. Finally, current challenges in the synthesis and application of PPMs using the CVD method are highlighted with suggested areas for future research.

## 2. CVD Method

CVD is a process for producing solid products from gases, and is a mature, lost cost, high efficiently technology for fabricating various kinds of semiconductor materials. CVD equipment mainly includes gas source control unit, deposition reaction chamber, deposition temperature control unit and vacuum exhaust and pressure control unit, and some experimental devices also have enhanced excitation energy control components. Therefore, according to the heating mode classification, it can be divided into thermal activation (resistance, high-frequency induction or infrared radiation heating, etc.), plasma enhancement, laser enhancement, microwave plasma enhancement, and other deposition modes, as shown in Table 1. In accordance with the reaction pressure classification, CVD can be divided into atmospheric pressure CVD (APCVD), low-pressure CVD (LPCVD) and ultrahigh vacuum CVD (UHVCVD, <10^−6^ Pa). According to gas phase classification, CVD can be further divided into Metal organic chemical vapor deposition (MOCVD), AACVD, direct liquid injection CVD (DLICVD), and HPCVD [40,41,42,43,44,45].

**Figure 2:**
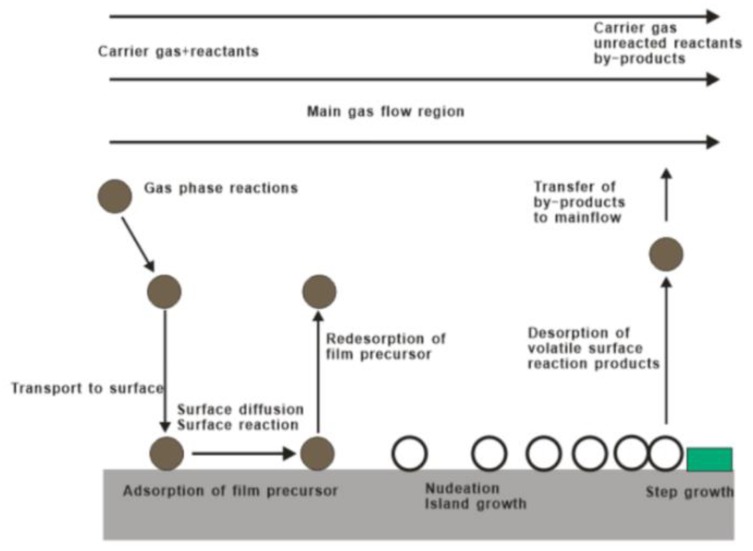
Basic reaction diagram of chemical vapor deposition.

Generally speaking, the reaction process of CVD mainly includes these parts: vapor-based reagents generation process, reactant transport process, chemical reaction process and reaction by-product removal process, as shown in Figure 2. Controllable variables of CVD method include gas flow rate and composition, deposition temperature, pressure, vacuum chamber shape, deposition time, and substrate material. In particular, basis of the relation between the gas diffusion constant, growth temperature, and gas pressure, the gas diffusion rate can be effectively tuned by an optimal combination of temperature and pressure. As such, CVD has the advantages of precise composition control, complete crystal deposition of thin films, large size and multi-substrate deposition, and deposition on complex substrates [40,41,42,43,44,45].

## 3. Synthesis and Application of PPMs Using the CVD Methods

In the past 10 years, the perovskite photovoltaic film and low dimensional materials and their constructed devices have become a hot topic. Great progress has been made in the preparation and optimization of materials and devices. However, there are still having many problems (such as poor stability, poor repeatability, toxicity, and so on) and challenges to solve. In this part, we aim to clarify why CVD technology can improve the properties of perovskite photovoltaic thin film materials, especially the stability and structure design of the materials, so as to optimize the performance of related devices.

### 3.1. Perovskite Photovoltaic Film Synthesis through a Variety of CVD Technologies

#### 3.1.1. Atmospheric Pressure and Low Pressure CVD (APCVD and LPCVD) Method

APCVD is a promising approach for producing high quality and large area film materials. In the last few years, one or two step APCVD methods have been used to fabricate perovskite films [46,47,48,49,50,51,52]. In 2015, one-step CVD method was used to fabricate planar heterojunction PSCs. By systematically optimizing the CVD parameters, such as temperature and growth time, high quality perovskite films of CH_3_NH_3_PbI_3_ and CH_3_NH_3_PbI_3−x_Cl_x_ were obtained. The solar power conversion efficiency (PCE) can be up to 11.1% [46]. At the same year, a two-step sequential tubular CVD (STCVD) method was first employed to fabricate MAPbI_3_ (MA=CH_3_NH_3_) material under open-air conditions and PSCs [47]. The measurement results showed that the device performance via STCVD method is significantly enhanced comparing with that of by the other methods of synthesis. For instance, the device Voc can be boost from 0.915 V to 1.001 V, with an impressive enhancement of 86 mV. The best PCE is greatly improved from 10.29% to 13.76%. That is to say, the STCVD method shows high potential to be applied in the commercial production of PSCs, due to the characteristics of high efficiency, stable feature, and low cost. Since then, atmospheric CVD technology has been used to prepare various perovskite films, such as CsFAPbI_3_, CsFAPbIBr, HC(NH_2_)_2_PbI_3x_Cl_x_, and so on [48,49,50,51,52]. These reported works prove that APCVD is a promising technique because it uses a scalable vapor based growth process and the resultant modules maintain a high steady state power at larger areas when compared to modules grown by a reference solution process [22,23,24].

**Figure 3:**
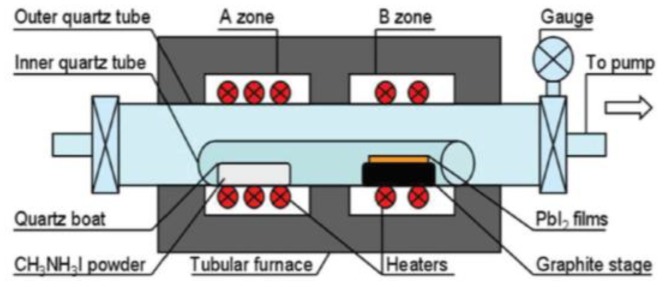
Schematic diagram of LPCVD instrument. Reproduced with permission from reference [34].

LPCVD are important thin film deposition techniques based on adsorption and subsequent surface reactions of precursor molecules in a high vacuum environment [53]. The schematic diagram of LPCVD instrument is shown in Figure 3. LPCVD is first introduced into fabrication of MAPbI_3_ perovskites by Luo group in 2015 [34]. Experimental results show that the prepared MAPbI_3_ films under low pressure have good crystallization, strong absorption, high stability, and long carrier diffusion length. Uniform and well-defined MAPbI_3_ layers are observed to be fully covered on the substrates, with a roughness of 19.6 nm and grain size up to 500 nm. Next year, Chen et al. have developed a different one-zone LPCVD to fabricate perovskites [54]. In this work, MAI and PbI_2_ are both loaded on a capped graphite boat, and then reacted at 120 °C for 60 or 120 min under a pressure of 133.3 Pa In this way, efficient PSCs of perovskite modules with a PCE of 6.22% on an active area of 8.4 cm^2^ are obtained. In addition, the crucial dependence of working pressure on the film formation is also revealed. Then, Qi group synthesize the FAPbI_3_ using the CVD with a pressure of ~100 Pa and a flow of drying nitrogen [55,56]. Cui group demonstrated an organic cation exchange concept for the preparation of high-quality a-FAPbI_3_ films using the CVD method under 10^−2^ Pa [57]. The PCE of the device was 12.4% based on the mesoscopic structure with no hysteresis. Since then, various PMs such as CH_3_NH_3_PbI_3_, (CH_3_NH3)_3_Bi_2_I_9_, and CsPbBr_3_ have been successfully prepared in low-pressure or ultra-low-pressure CVD systems [58,59,60,61,62,63,64,65,66,67]. The published works in recent years using the LPCVD method to fabricated perovskite materials are summarized and listed in Table 2.

#### 3.1.2. Aerosol Assisted CVD (AACVD) Method

Compared with other deposition methods, AACVD is a low-cost and scalable technique. AACVD process occurs under ambient pressure and requires moderately volatile precursors, which should be a very attractive film fabrication technology and has a good potential for scale-up. Lewis et al. first use the AACVD method to the fabrication of MAPbBr_3_ perovskite film in 2014 [68]. The dilute nebulized precursors are transported by N_2_ gas into the tubular reaction furnace. The yellow perovskite film is deposited at 250 °C. Subsequently, phase pure, compositionally uniform MAPbI_3_ films on large glass substrate are prepared by Palgrave in 2015 using the AACVD method [69]. Then, several research groups have developed a one-step AACVD method to deposit perovskite thin films [70,71]. For instance, Liu et al. present a novel NH_4_Cl + CH_3_NH_3_I mixing vapor-assisted CVD method to realize the low-temperature and rapid preparation of perovskite layers [71].

Next year, Binions et al. develop a two-step sequential AACVD method to deposit MAPbI_3_ film [72]. To obtain uniform and thick enough perovskite film, a modified three-step AACVD method was proposed by Afzal et al. [73]. In the three-step AACVD deposition technique, a cold-walled horizontal-bed AACVD reactor was used to deposit perovskite MAPbI_3_ film. To overcome the solubility limitation of bromide in conventional polar solvent, a novel Br_2_-vapor-assisted CVD method was elaborately design to realize the fast anion-exchange from CsPbI_3_ to CsPbBr_3_, and thus structural stability of inorganic perovskites is enhanced accordingly [74]. PSCs based on these perovskite materials demonstrate good long term stability and give ~90% of initial efficiencies even after 21 days of exposure to air.

#### 3.1.3. Hybrid Physical CVD (HPCVD) Method

In 2014, Qi et al. first introduced HPCVD technology for the effective deposition of a perovskite layer [40]. In this method, PbX_2_ was evaporated in a high-vacuum environment to obtain uniform films, and methyl ammonium iodide (MAI) was then heated to 185 °C and transported into the reaction site by N_2_ gas to fabricate perovskite solar cells. Next year, high-quality CH_3_NH_3_PbI_3_ films and corresponding solar cells were prepared by HPCVD method with a reaction temperature of 100 °C [75]. By optimizing the reaction temperature and pressure, efficient CH_3_NH_3_PbI_3_ solar cells were fabricated with high conversion efficiency up to 12.3%. In the same year, Peng etc. [76] also synthesized the high-quality CH_3_NH_3_PbI_3_ films using the HPCVD process in a vacuum and isothermal environment. CH_3_NH_3_PbI_3_ solar cells with high PCE up to 14.7% were fabricated at 82 °C. In 2016, all-vacuum processed methyl ammonium lead halide perovskite by a sequence of physical vapor deposition of PbI_2_ and CVD of CH_3_NH_3_I under a static atmosphere was fabricated [77,78]. A dependence of residual PbI_2_ on the solar cells performance is displayed, while photovoltaic devices with efficiency up to 11.6% were achieved. It should be pointed out that, the diffusion rate can be varied over a wide range by controlling its gas pressure when the growth temperature is limited to reasonable value for the HPCVD system. Thus, high-quality perovskite films with fully covered, smooth surfaces are achieved by using the HPCVD method [37,79]. A hybrid CVD with cation exchange method is developed for preparation of Cs-substituted mixed cation perovskite films [80,81,82]. This technique shows a high potential toward scaling-up the Cs-substituted perovskite from lab solar cell scale 1.5 cm^2^ to module scale 5 cm^2^ with a high module PCE of 14.6% (12.0 cm^2^ active areas). Cs_0.07_FA_0.93_PbI_3_ PSC prepared by this method shows 14.0% PCE after 1200 min steady-state measurement, demonstrating the promising device stability achieved by this perovskite fabrication technique [83].

### 3.2. Low Dimensional Perovskite Photovoltaic Materials Synthesized by CVD Technologies

Over the last five years, there has been tremendous progress in the development of nanoscale PPMs which possess a wide range of band gaps and tunable optical and electronic properties. These excellent photoelectric properties and ultra-high density of nanostructured PPMs can serve as ideal candidates for [81,82,83,84,85,86,87,88,89] solar cells.

The emerging 2D PPMs are attracting more interest due to the long charge carrier lifetime, high photoluminescence quantum efficiency, and great defect tolerance [90]. Recently, Ha et al. first developed CVD synthesis of perovskite nanoplatelets on mica substrate [20]. This method was further employed by Su group [91] to push the ample thickness (below 10 nm) on mica substrates. The weak material–substrate interaction and low cohesive energy of the perovskite lead to the growth of large-scale ultrathin 2D crystals. Wang et al. [92] used a dual precursor CVD method to grown MAPbCl_3_ 2D sheet with a thickness of 8.7 nm and lateral dimension of over 20 mm. Nanosheets with thicknesses as low as 1.3 nm have been synthesized using CVD at a low pressure of 40 Torr and 120 °C. Bao and other research teams [21,93,94,95] use the CVD to create 2D CH_3_NH_3_PbI_3_ perovskites. Lan et al. show that process pressure can be used to grow PbI_2_ with high crystallinity on roughened surfaces [96]. CVD was also used to convert the as-synthesized nanosheets to CH_3_NH_3_PbI_3_. Chen et al. synthesized ultrathin (BA)_2_(MA)_n−1_PbnBr_3n+1_ perovskites with thickness down to 4.2 nm and lateral dimension up to 57 mm [97]. These results suggest that the one-step and two-step vapor phase synthetic approaches are powerful in prepare low dimensional PMs.

Particularly, thin 2D materials can be used as hole extraction layers in organolead halide PSCs [98,99]. MoS_2_ and WS_2_ layers with a polycrystalline structure were synthesized by a thermal CVD system at 950 °C and 1 Torr pressure. The PCE of the MoS_2_- and WS_2_-based PSCs were 9.53% and 8.02%, respectively. These results suggest that 2D materials such as MoS_2_ and WS_2_ can be promising candidates for the formation of hole extraction layers in the PSCs. FAPb(Br_x_I_1−x_)_3_ nanoplatelets with gradient bandgap are fabricated using CVD method [100]. During the whole vapor conversion process, CVD furnace temperature was 140 °C, and pressure was about 500 mTorr with a controlled Ar gas flow rate of 35 sccm (standard cubic centimeters per minute) and reaction time 2 h.

## 4. Electrode, Window, Blocking and Electron Transport Layer Materials of Perovskite Devices Prepared by CVD Technologies

Interfacing perovskites with other 1D or 2D materials including graphene, carbon nanotubes (CNTs), SnO_2_, ZnO, CuO_x_, and CdS significantly broadens the application range of the perovskite materials. Therefore, using various CVD technologies to prepare high-quality electrode, window, blocking, and electron transport layer materials of perovskite devices can effectively promote device design and enhances the performance of the functional devices.

### 4.1. APCVD Method

**Figure 4:**
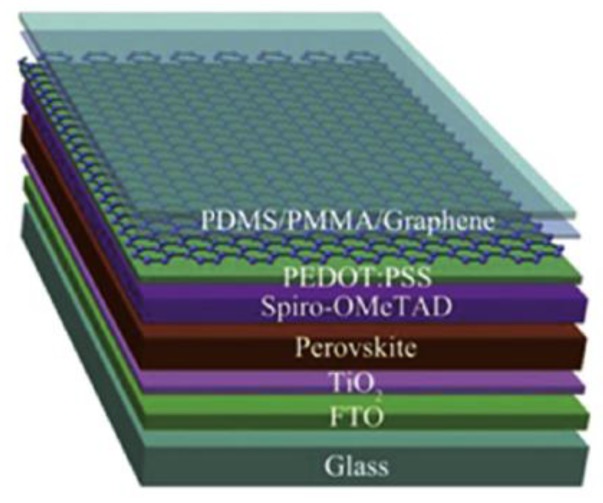
Schematic diagram of the semitransparent PSC using the graphene as top electrode. Reproduced with permission from reference [105].

CVD synthesized graphene films can be used as electrodes in normal structure PSCs to replace the traditional conductive fluorine-doped or indium tin oxide (FTO or ITO) electrodes, either on rigid substrates or on flexible substrates [101,102,103,104,105,106,107,108,109,110,111,112,113,114,115]. Luo et al. [101] prepared flexible PSCs with a PCE of 11.9% using the graphene as bottom electrode and CNTs as top electrode. Meng et al. [102] synthesized the high quality graphene using CVD method as bottom electrodes in normal structure PSC. An efficiency of 13.93% was obtained from the reverse scan of the current density–voltage (J–V) curves. In 2015, Sung et al. [103] first reported the adoption of graphene as bottom electrodes in inverted structure PSCs. In 2016, Liu et al. [104] also successfully fabricated the flexible PSCs with double layer graphene films as bottom electrodes. The reasonable transparency is ~90% in the visible range, which is much higher than that of ITO electrode [105,106]. The semitransparent PSC using the graphene as top electrode is shown in Figure 4. The following year, Yoon and his collaborators [107] reported super-flexible PSCs by using MoO_3_-modified single-layer CVD graphene as bottom electrodes with a maximum PCE of 16.8%. The graphene-based flexible PSCs can maintain over 90% of the original efficiencies after bending test of 1000 cycles at a bending radius of 2 mm. In order to attain higher efficiencies of the graphene-based PSCs, how to further reduce the sheet resistance of graphene films is a key problem. Im and his coworkers employed AuCl_3_-doped graphene [116,117,118] and amide TFSA-doped graphene [109] as bottom electrodes to make super-flexible PSCs. After highly p-type chemical doping with two dopants, the sheet resistance of single layer graphene film decreased significantly to approximately 100 Ω sq^−1^, which is much lower than the monolayer graphene. Lang et al. [110] implemented large-area CVD graphene films to obtain semitransparent PSCs and achieving a champion PCE of 13.2% in the final tandem cells. Zhou et al. [111] successfully fabricated terminal perovskite/Si tandem PSCs with a high efficiency of 18.1%. In addition, graphene can also be used as electron transport and blocking layer [112]. Single layer graphene was introduced into PSCs as an air and metal blocking layer to protect the perovskite layer, because the graphene is electrically conductive and can block the metal ions, oxygen, and water into the perovskite layer. Therefore, PSCs including a graphene layer showed a significantly enhanced stability under ambient conditions or thermal annealing process [113]. Therefore, we can see it clearly that the conductivity and quality of the transferred graphene films can severely impact the device performance of PSCs.

Except the graphene, CNTS (network films) prepared by CVD method are also used as electrodes of perovskite devices [114,115]. CNT network films were synthesized using the floating catalyst CVD method using a tube furnace. The transparent CNTs top electrode acts as hole collecting layer and light transmission. The PSCs with a PCE up to 8.31% has been achieved. The published works of using the graphene and CNT materials as top and bottom electrodes are summarized and listed in Table 3.

### 4.2. PECVD Method

Large area and functional TiO_2−x_ films using as the hole blocking electron transport layers in PSC architectures are synthesized by roll to roll PECVD system [116]. The PECVD system has a dual laminar flow design to provide two reaction zones to improve net growth rate and uniformity and minimize the entrainment of surrounding air. Each reaction zone incorporates a dielectric barrier system to provide plasma activation and extraction of waste products to avoid contamination. The film thickness can be effectively controlled by the number of passes under the coating head. The experimental results showed that PECVD is capable for producing 50 nm TiO_2−x_ layers with sufficient quality and uniformity for use in perovskite based photovoltaic devices and increasing cell efficiency further.

### 4.3. Pulsed CVD Method

Pulsed CVD was first used by Bush et al. to prepare SnO_2_/ZTO window layer of perovskite/silicon tandem solar cells devices [117]. The vapor processes can produce a compact, uniform, and highly transparent SnO_2_/ZTO bilayer with efficient hole-blocking ability and sputter buffer layer properties. The devices have a good thermal and ambient stability compared with the standard encapsulation such as glass. The perovskite cells were coupled with silicon heterojunction bottom cells and ITO layers. The resulting tandem reached an efficiency of 23.6% with no hysteresis. The maximum power can be stabilized over more than 30 minutes under illumination.

Then, in order to solve the problems of high thermal mismatch and low fracture level of PSCs and improve the conversion efficiency of the devices, Cheacharoen et al. prepared the 4 nm SnO_2_ and 2 nm Zinc oxide tin layer by pulsed CVD method at 100 °C [118]. The stabilities of the encapsulated PSCs were measured by the International Electrotechnical Commission (IEC) 61646 standard test of 200 temperature cycles from −40 °C to 85 °C and observed no visible delamination and less than a 10% change in performance.

### 4.4. Physical Chemical Vapor Deposition (PCVD) Method

CdS nanorods arrays and thin film as an electron transport layer in PSCs are fabricated by PCVD method [119,120]. A parametric optimization of the CdS layer thickness and an advantageous in situ Cd-doping of the perovskite thin film lead to a high PCE of ~14.68%. In particular, this lower synthesis temperature (<85 ℃) is convenient for implementation a flexible PSCs directly upon plastic PET substrate. Then, 3D architecture PSCs using the CdS nanorods arrays as an electron transport layer were designed and prepared via a layer-by-layer PCVD process. The CdS NRs not only provided a scaffold to the perovskite film, but also increased the interfacial contact between the perovskite film and electron transport layer. As an optimized result, a high PEC of 12.46% with a short-circuit current density of 19.88 mA/cm^−2^, an open-circuit voltage of 1.01 V was obtained.

### 4.5. AACVD Method

As mentioned earlier, CNT film has shown to be very effective in replacing metal electrodes and enhancing the stability of PSCs in air environment. Randomly oriented CNT networks with high purity and long nanotube bundle length are synthesized by the AACVD method [121]. Then the carbon-sandwiched perovskite device was studied by using the CVD synthesized CNT on top of MAPbI_3_. Characterization results show that the encapsulated device (with a structure ITO/C_60_/MAPbI_3_/CNT) shows high stability against both air and light, with around 90% of the initial efficiency after 2000 h under actual operation conditions.

As a variant of conventional CVD process, AACVD allows for the use of non-volatile metal precursors as the limiting factor is soluble substance rather than volatile substance. Furthermore, the AACVD growth rate could reduce to one comparable or lower than that of atomic layer deposition, making it attractive for ultra-thin film growth. In 2018, ultrathin and compact ZnO films have been successfully deposited on FTO electrodes via AACVD method [122]. ZnO films were carried out in a horizontal bed cold-walled tubular reactor with a size 170 × 60 mm. The deposition temperature was set at 350 °C and the reaction time was 8 mins. After deposition, samples were annealed in a tube furnace at 500 °C for 1 hour under argon atmosphere. Planar PSCs with conventional material CH_3_NH_3_PbI_3_ were fabricated under ambient conditions and best PCE of 11.75% was achieved. Also last year, ultra-thin CuOx coatings using as a hole extraction layer in inverted PSCs was prepared by AACVD technique [123]. The resulting CH_3_NH_3_PbI_3_ PSC achieves a maximum PCE of 8.26%, demonstrating the efficient hole transporting ability in the CuO_x_ coatings.

## 5. Conclusions and Future Outlook

In summary, we have made a review on the preparation and application of perovskite photovoltaic and related device materials by a variety of CVD technologies—including APCVD, LPCVD, HPCVD, HCVD, AACVD, PECVD, and pulsed CVD—and the most recent progress was highlighted over the past five years. Perovskite photovoltaic materials and related device properties with different compositions, morphologies, structures, and dimensions are all discussed. Obviously, it is observed that high stability, high controllability, and high scalability PPMS and high performance devices could be achieved by CVD method through controlling gas flow rate, growth temperature, pressure, and reaction time of the reaction systems. For example, high efficient PSCs with a PCE 23.6% have been achieved by utilizing CVD method to prepare needed materials. Thus, CVD methods owning high stability, low-cost, and scalable features are one of the best choices to prepare high quality perovskite photovoltaic materials, which could be applied for potential industry applications of building PSCs with high efficiency. 

However, just as impressive as the progress has been, there are novel opportunities and challenges remaining:

1. Systematic study of synthesis mechanism of CVD method

There is no doubt that great progress has been made in the preparation and optimization of PPMS and devices using various CVD technologies. However, scientists are mainly concerned about how to realize materials and devices with CVD method, and lack of attention and in-depth research on the physical mechanism of synthesis. Understanding the mechanism behind the formation of these perovskite photovoltaic materials will help researchers to come up with effective strategies to combat the emerging challenges of this family of materials, such as stability under ambient conditions and toxicity, towards next generation applications in photovoltaics and optoelectronics.

2. Stabilization

A major limiting factor for the commercialization of perovskite photovoltaic devices is the low stability of the materials. They react very easily with water in the air and are easily oxidized by oxygen in the air. At the same time, the thermal stability of the material is poor, easy to decompose when heated. The longest reported stabilization time is now in the hundreds of days. The stability problems will be amplified at the nanoscale due to the relative surface area being much larger compared to the bulk materials. Thus, stabilizing of the materials and devices is critically important for the perovskites. Selecting ideal encapsulation and protection materials for the perovskites during the process of fabrication is important.

3. Novel perovskite structures

As we mentioned in the introduction, the perovskite structure takes the form of ABX_3_. Within this framework, lead and tin are the most commonly used in B position, resulting in their physical properties being unable to be tuned to a precise extent. Searching for novel complex perovskite structures may solve the problem. In particular, many groups have demonstrated that the optical and electrical properties of perovskite photovoltaic materials greatly depend on their dimensions. Thus, ability to fully manipulate their dimensions will be vital for understanding the structure–property–device behavior relationship.

4. Interfaces and heterojunctions

Low-dimensional perovskite materials possess large and well-defined surfaces, and such surfaces can interact strongly with another material’s surface when bringing these two materials together to form a heterojunction. Perovskite photovoltaic materials interfacing with low-dimensional graphene, CNTs, SnO_2_, ZnO, CuO_x_, and CdS materials using the CVD method have been proved can significantly broadens the application and enhances the performance of the functional devices. However, directly growing a lateral heterojunction along the edge of the perovskite low-dimensional materials remains challenging. More complicated heterojunctions with better controlled structures, compositions, and performance are desired in the near future.

CVD technology has shown some advantages and potential applications in preparing perovskite photovoltaic materials and devices than other synthetic methods. It could benefit from the controllable and optimizable growth temperature, pressure, and the moderate gas-phase reaction rate in CVD process. Therefore, the continued development of cost-effective CVD technology is crucial to further commercialization of perovskite photovoltaic devices. This method must draw deep attention from a wide range of researchers and we also hope that it could be vigorously promoted in perovskite materials and devices.

## Figures and Tables

**Table 1:** Different CVD devices, heating mode and basic schematic diagram ^1^.

Device Form	Heating Method (Temperature Range, °C)	Principle Diagram
Tubular furnace type	Resistance heating mode (~1000)	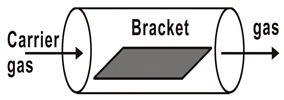
Vertical type	Plate heating mode (~500) Induction heating mode (~1200)	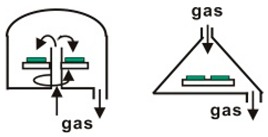
Cylinder type	Induction heating mode (~1200) Infrared radiation heating mode (~1200)	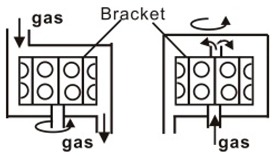
Tandem surround type	Plate heating mode (~500) Infrared radiation heating mode (~1200)	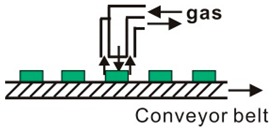

^1^ Referrence [45].

**Table 2:** Perovskite materials were synthesized by LPCVD technique.

Perovskite Material	Pressure	PCE (%)	Ref.
CH_3_NH_3_PbI_3_	1 Torr	14.99 (Mesoscopic)	[54]
		15.37 (Planar)	
FAI (Formamidinium iodide)	2 × 10^−2^ Pa	14.2	[55]
CH_3_NH_3_PbI_3_	1 × 10^−3^ Pa	15.6	[56]
α-FAPbI_3_	10^−2^ Pa	12.4	[57]
AMX_3_	10^−5^–10^−6^ mbar	~	[58]
CH_3_NH_3_PbBrI_3-x_	170 Torr	~	[59]
CH_3_NH_3_I(MAI)	~	16.42	[60]
CH_3_NH_3_PbI_3_	10 hPa	~	[61]
CsPbX_3_ (X = Cl, Br, I)	4.8(4.8,5.2) Torr	5.9 (10.0, 8.3)	[62]
(CH_3_NH_3_)_3_Bi_2_I_9_	10^−6^ hPa	0.047	[63]
CH_3_NH_3_PbI_3_	10^4^ Pa	~	[64]
CH_3_NH_3_PbI_3_	1 Torr	7.9	[65]
CsPbBr_3_	150 Pa	~	[66]
(CH_3_NH_3_)_3_Bi_2_I_9_	10 hpa	0.047	[67]

**Table 3:** Summary of the PSCs with graphene and CNTs electrodes.

Electrode Material	Device Structures	PCE (%)	Ref.
Graphene CNTS	FET/Graphene/TiO_2_/PCBM/MAPbI_3_/Spiro-OMeTAD/CNTs	11.9	[101]
Graphene	Quartz/graphene/C_60_/MAPbI_3_/carbon	13.93	[102]
Graphene	Glass/graphene/MoO_3_/PEDOT:PSS/MAPbI_3_/C_60_/BCP/LiF/Al	17.1	[103]
Graphene	PET/ZEOCOAT/graphene/P_3_HT/MAPbI_3_/PC71BM/Ag	11.5	[104]
Graphene	FTO/TiO_2_/MAPbI3-xClx/Spiro-OMeTAD/PEDOT:PSS/graphene	12.37	[105]
Graphene	PET/graphene/PEDOT:PSS/MAPbI_3_/PCBM/Al	13.94	[106]
Graphene	PEN/graphene/MoO_3_/PEDOT:PSS/MAPbI_3_/C_60_/BCP/LiF/Al	16.8	[107]
Graphene	PET/graphene/PEDOT:PSS/FAPbI_3−_xBrx/PCBM/Al	17.9	[108]
Graphene	Glass or PDMS/graphene/PEDOT:PSS/FAPbI_3−_xBrx/PCBM/Al	18.3	[109]
Graphene	FTO/TiO_2_/MAPbI_3_/Spiro-OMeTAD/graphene	6.2	[110]
Graphene	FTO/TiO_2_/MAPbI_3−_xClx/Spiro-OMeTAD/PEDOT:PSS/graphene	11.8	[111]
CNTs	Tifoil/CH_3_NH_3_PbI_3_/TiO_2_NTs/Spiro-OMeTAD/CNTs	8.31	[114]
CNTs	Glass/FTO/TiO_2_/CH_3_NH_3_PbI_3_/CNTs	3.88	[115]

## References

[1] Rose G. (1839). Beschreibung einiger neuen Mineralien des Urals. Ann. Phys..

[2] Liu M.Z., Johnston M.B., Snaith H.J. (2013). Efficient planar heterojunction perovskite solar cells by vapour deposition. Nature.

[3] Lee M.M., Teuscher J., Miyasaka T., Murakami T.N., Snaith H.J. (2012). Efficient hybrid solar cells based on meso-superstructured organometal halide perovskites. Science.

[4] Stranks S.D., Eperon G.E., Grancini G., Menelaou C., Alcocer M.J., Leijtens T., Herz L.M., Petrozza A., Snaith H.J. (2013). Electron-hole diffusion lengths exceeding 1 micrometer in an organometal trihalide perovskite absorber. Science.

[5] Liu D., Kelly T.L. (2014). Perovskite solar cells with a planar heterojunction structure prepared using room-temperature solution processing techniques. Nat. Photonics.

[6] Jang D.M., Park K., Kim D.H., Park J., Shojaei F., Kang H.S., Ahn J.P., Lee J.W., Song J.K. (2015). Reversible halide exchange reaction of organometal trihalide perovskite colloidal nanocrystals for full-range band gap tuning. Nano Lett..

[7] Dong R., Fang Y., Chae J., Dai J., Xiao Z., Dong Q., Yuan Y., Centrone A., Zeng X.C., Huang J. (2015). High-gain and low-driving-voltage photodetectors based on organolead triiodide perovskites. Adv. Mater..

[8] Veldhuis S.A., Boix P.P., Yantara N., Li M., Sum T.C., Mathews N., Mhaisalkar S.G. (2016). Perovskite materials for light-emitting diodes and lasers. Adv. Mater..

[9] Shan Q.S., Song J.Z., Zou Y.S., Li J.H., Xu L.M., Xue J., Dong Y.H., Han B.N., Chen J.W., Zeng H.B. (2017). High performance metal halide perovskite light-emitting diode: From material design to device optimization. Small.

[10] Ha S.T., Su R., Xing J., Zhang Q., Xiong Q.H. (2017). Metal halide perovskite nanomaterials: Synthesis and applications. Chem. Sci..

[11] Makarov S., Furasova A., Tiguntseva E., Hemmetter A., Berestennikov A., Pushkarev A., Zakhidov A., Kivshar Y. (2019). Halide-perovskite resonant nanophotonics. Adv. Opt. Mater..

[12] Dong Q., Fang Y., Shao Y., Mulligan P., Qiu J., Cao L., Huang J. (2015). Electron-hole diffusion lengths > 75 μm in solution-grown CH_3_NH_3_PbI_3_ single crystals. Science.

[13] Nie W., Tsai H., Asadpour R., Blancon J.C., Neukirch A.J., Gupta G., Crochet J.J., Chhowalla M., Tretiak S., Alam M.A. (2015). High-efficiency solution-processed perovskite solar cells with millimeter-scale grains. Science.

[14] Xiao Z., Bi C., Shao Y., Dong Q., Wang Q., Yuan Y., Wang C., Gao Y., Huang J. (2014). Efficient, high yield perovskite photovoltaic devices grown by interdiffusion of solution-processed precursor stacking layers. Energy Environ. Sci..

[15] Ning Z., Gong X., Comin R., Walters G., Fan F., Voznyy O., Yassitepe E., Buin A., Hoogland S., Sargent E.H. (2015). Quantum-dot-in-perovskite solids. Nature.

[16] Deng W., Xu X.Z., Zhang X.J., Zhang Y.D., Jin X.C., Wang L., Lee S.T., Jie J.S. (2016). Organometal halide perovskite quantum dot light-emitting diodes. Adv. Funct. Mater..

[17] Horvath E., Spina M., Szekrenyes Z., Kamaras K., Gaal R., Gachet D., Forro L. (2014). Nanowires of methylammonium lead iodide (CH_3_NH_3_PbI_3_) prepared by low temperature solution-mediated crystallization. Nano Lett..

[18] Petrov A.A., Pellet N., Seo J.Y., Belich N.A., Kovalev D.Y., Shevelkov A.V., Goodilin E.A., Zakeeruddin S.M., Tarasov A.B., Graetzel M. (2017). New insight into the formation of hybrid perovskite nanowires via structure directing adducts. Chem. Mater..

[19] Liu P., He X., Ren J., Liao Q., Yao J., Fu H. (2017). Organic–Inorganic hybrid perovskite nanowire laser arrays. ACS Nano.

[20] Ha S.T., Liu X.F., Zhang Q., Giovanni D., Sum T.C., Xiong Q.H. (2014). Synthesis of organic–Inorganic lead halide perovskite nanoplatelets: Towards high-performance perovskite solar cells and optoelectronic devices. Adv. Opt. Mater..

[21] Liu J.Y., Xue Y.Z., Wang Z.Y., Xu Z.Q., Zheng C.X., Weber B., Song J.C., Wang Y.S., Lu Y.R., Zhang Y.P. (2016). Two-dimensional CH_3_NH_3_PbI_3_ perovskite: Synthesis and optoelectronic application. ACS Nano.

[22] Burschka J., Pellet N., Moon S.J., Humphry-Baker R., Gao P., Nazeeruddin M.K., Grätzel M. (2013). Sequential deposition as a route to high-performance perovskite-sensitized solar cells. Nature.

[23] Zhou H.P., Chen Q., Li G., Luo S., Song T.B., Duan H.S., Hong Z.R., You J.B., Liu Y.S., Yang Y. (2014). Interface engineering of highly efficient perovskite solar cells. Science.

[24] Eperon G.E., Burlakov V.M., Docampo P., Goriely A., Snaith H.J. (2014). Morphological control for high performance, solution-processed planar heterojunction perovskite solar cells. Adv. Funct. Mater..

[25] Dualeh A., Tetreault N., Moehl T., Gao P., Nazeeruddin M.K., Grätzel M. (2014). Effect of annealing temperature on film morphology of organic–inorganic hybrid perovskite solid-state solar cells. Adv. Funct. Mater..

[26] Xiao M., Huang F., Huang W., Dkhissi Y., Zhu Y., Etheridge J., Weale A.G., Bach U., Cheng Y.B., Spiccia L. (2014). A fast deposition-crystallization procedure for highly efficient lead iodide perovskite thin-film solar cells. Angew. Chem. Int. Ed..

[27] Chen C.W., Kang H.W., Hsiao S.Y., Yang P.F., Chiang K.M., Lin H.W. (2014). Efficient and uniform planar-type perovskite solar cells by simple sequential vacuum deposition. Adv. Mater..

[28] Longo G., Gil-Escrig L., Degen M.J., Sessolo M., Bolink H.J. (2015). Perovskite solar cells prepared by flash evaporation. Chem. Commun..

[29] Deng Y., Peng E., Shao Y., Xiao Z., Dong Q., Huang J. (2015). Scalable fabrication of efficient organolead trihalide perovskite solar cells with doctor-bladed active layers. Energy Environ. Sci..

[30] Hwang K., Jung Y.S., Heo Y.J., Scholes F.H., Watkins S.E., Subbiah J., Jones D.J., Kim D.Y., Vak D. (2015). Toward large scale roll-to-roll production of fully printed perovskite solar cells. Adv. Mater..

[31] Barrow A.T., Pearson A.J., Kwak C., Dunbar A.D.F., Buckley A.R., Lidzey D.G. (2014). Efficient planar heterojunction mixed-halide perovskite solar cells deposited via spray-deposition. Energy Environ. Sci..

[32] Wei Z., Chen H., Yan K., Yang S. (2014). Inkjet printing and instant chemical transformation of a CH_3_NH_3_PbI_3_/nanocarbon electrode and interface for planar perovskite solar cells. Angew. Chem. Int. Ed..

[33] Chen Q., Zhou H., Hong Z., Luo S., Duan H.S., Wang H.H., Liu Y., Li G., Yang Y. (2014). Planar heterojunction perovskite solar cells via vapor-assisted solution process. J. Am. Chem. Soc..

[34] Luo P.F., Liu Z.F., Xia W., Yuan C.C., Cheng J.G., Lu Y.W. (2015). Uniform, stable, and efficient planar-heterojunction perovskite solar cells by facile low-pressure chemical vapor deposition under fully open-air conditions. ACS Appl. Mater. Interfaces.

[35] Chen J., Zhou S., Jin S., Li H., Zhai T. (2016). Crystal organometal halide perovskites with promising optoelectronic applications. J. Mater. Chem. C.

[36] Ono L.K., Leyden M.R., Wang S., Qi Y. (2016). Organometal halide perovskite thin films and solar cells by vapor deposition. J. Mater. Chem. A.

[37] Luo P.F., Zhou S.W., Xia W., Cheng J.G., Xu C.X., Lu Y.W. (2017). Chemical vapor deposition of perovskites for photovoltaic application. Adv. Mater. Interfaces.

[38] Xu X., Wang W., Zhou W., Shao Z. (2018). Recent advances in novel nanostructuring methods of perovskite electrocatalysts for energy-related applications. Small Methods.

[39] Jamal M.S., Bashar M.S., Hasan A.M., Almutairi Z.A., Alharbi H.F., Alharthi N.H., Karim M.R., Misran H., Amin N., Sopian K.B. (2018). Fabrication techniques and morphological analysis of perovskite absorber layer for high-efficiency perovskite solar cell: A review. Renew. Sustain. Energy Rev..

[40] Leyden M.R., Ono L.K., Raga S.R., Kato Y., Wang S., Qi Y. (2014). High performance perovskite solar cells by hybrid chemical vapor deposition. J. Mater. Chem. A.

[41] Ting J.M., Huang N.Z. (2001). Thickening of chemical vapor deposited carbon fiber. Carbon.

[42] Darr J.A., Guo Z.X., Raman V., Bououdina M., Rehman I.U. (2004). Metal organic chemical vapour deposition (MOCVD) of bone mineral like carbonated hydroxyapatite coatings. Chem. Commun..

[43] Li Y., Mann D., Rolandi M., Kim W., Ural A., Hung S., Javey A., Cao J., Wang D., Yenilmez E. (2004). Preferential growth of semiconducting single-walled carbon nanotubes by a plasma enhanced CVD method. Nano Lett..

[44] Suresh A., Anastasio D., Burkey D.D. (2014). Potential of hexyl acrylate monomer as an initiator in photo-initiated CVD. Chem. Vapor Depos..

[45] Jones A.C., Hitchman M.L. (2009). Chemical Vapour Deposition: Precursors, Processes and Applications.

[46] Tavakoli M.M., Gu L.L., Gao Y., Reckmeier C., He J., Rogach A.L., Yao Y., Fan Z.Y. (2015). Fabrication of efficient planar perovskite solar cells using a one-step chemical vapor deposition method. Sci. Rep..

[47] Luo P.F., Liu Z.F., Xia W., Yuan C.C., Cheng J.Q., Xu C.X., Lu Y.W. (2015). Chlorine-conducted defect repairmen and seed crystal-mediated vapor growth process for controllable preparation of efficient and stable perovskite solar cells. J. Mater. Chem. A.

[48] Leyden M.R., Meng L.Q., Jiang Y., Ono L.K., Qiu L.B., Juarez-Perez E.J., Qin C.J., Adachi C.H., Qi Y.B. (2017). Methylammonium lead bromide perovskite light-emitting diodes by chemical vapor deposition. J. Phys. Chem. Lett..

[49] Wei Q., Bi H., Yan S., Wang S.W. (2018). Morphology and interface engineering for organic metal halide perovskite-based photovoltaic cells. Adv. Mater. Interfaces.

[50] Sun J.C., Wu J., Tong X., Wang Y.N., Wang Z.M. (2018). Organic/inorganic metal halide perovskite optoelectronic devices beyond solar cells. Adv. Sci..

[51] Tavakoli M.M., Zakeeruddin S.M., Grätzel M., Fan Z.Y. (2018). Large-grain tin-rich perovskite films for efficient solar cells via metal alloying technique. Adv. Mater..

[52] Swartwout R., Hoerantner M.T., Bulović V. (2019). Scalable deposition methods for large-area production of perovskite thin films. Energy Environ. Mater..

[53] Park N.G. (2016). Crystal growth engineering for high efficiency perovskite solar cells. CrystEngComm.

[54] Shen P.S., Chen J.S., Chiang Y.H., Li M.H., Guo T.F., Chen P. (2016). Low-pressure hybrid chemical vapor growth for efficient perovskite solar cells and large-area module. Adv. Mater. Interfaces.

[55] Leyden M.R., Lee M.V., Raga S.R., Qi Y.B. (2015). Large formamidinium lead trihalide perovskite solar cells using chemical vapor deposition with high reproducibility and tunable chlorine concentrations. J. Mater. Chem. A.

[56] Leyden M.R., Jiang Y., Qi Y. (2016). Chemical vapor deposition grown formamidinium perovskite solar modules with high steady state power and thermal stability. J. Mater. Chem. A.

[57] Zhou Z.M., Pang S.P., Ji F.X., Zhang B., Cui G.L. (2016). The fabrication of formamidinium lead iodide perovskite thin films via organic cation exchange. Chem. Commun..

[58] Ávila J., Momblona C., Boix P.P., Sessolo M., Bolink H.J. (2017). Vapor-deposited perovskites: The route to high-performance solar cell production?. Joule.

[59] Wang Y.P., Chen Z.Z., Deschler F., Sun X., Lu T.M., Wertz E.A., Hu J.M., Shi J. (2017). Epitaxial halide perovskite lateral double heterostructure. ACS Nano.

[60] Tong G.Q., Lan X.Z., Song Z.H., Li G.P., Li H., Yu L.W., Xu J., Jiang Y., Sheng Y., Shi Y. (2017). Surface-activation modified perovskite crystallization for improving photovoltaic performance. Mater. Today Energy.

[61] Stümmler D., Sanders S., Pfeiffer P., Weingarten M., Vescan A., Kalisch H. (2017). Direct chemical vapor phase deposition of organometal halide perovskite layers. MRS Adv..

[62] Guo P.F., Hossain M.K., Shen X., Sun H.B., Yang W.C., Liu C.P., Ho C.Y., Kwok C.K., Tsang S.W., Luo Y.S. (2018). Room-temperature red-green-blue whispering-gallery mode lasing and white-light emission from cesium lead halide perovskite (CsPbX_3_, X = Cl, Br, I) microstructures. Adv. Opt. Mater..

[63] Stümmler D., Sanders S., Pfeiffer P., Wickel N., Simkus G., Heuken M., Baumann P.K., Vescan A., Kalisch H. (2018). Investigation of perovskite solar cells employing chemical vapor deposited methylammonium bismuth iodide layers. MRS Adv..

[64] Lei Y., Gu L.Y., Yang X.G., Zhang L.L., Gao Y.H., Li J.X., Zheng Z. (2018). Fast chemical vapor-solid reaction for synthesizing organometal halide perovskite array thin films for photodetector applications. J. Alloys Compd..

[65] Tran V.D., Pammi S.V.N., Dao V.D., Choi H.S., Yoo S.G. (2018). Chemical vapor deposition in fabrication of robust and highly efficient perovskite solar cells based on single-walled carbon nanotubes counter electrodes. J. Alloys Compd..

[66] Tian C.C., Wang F., Wang Y.P., Yang Z., Chen X.J., Mei J.J., Liu H.Z., Zhao D.X. (2019). Chemical vapor deposition method grown all-inorganic perovskite microcrystals for self-powered photodetectors. ACS Appl. Mater. Interfaces.

[67] Stümmler D., Sanders S., Gerstenberger F., Pfeiffer F., Simkus G., Baumann P.K., Heuken M., Vescan M., Kalisch H. (2019). Reaction engineering of CVD methylammonium bismuth iodide layers for photovoltaic applications. J. Mater. Res..

[68] Lewis D.J., O’Brien P. (2014). Ambient pressure aerosol-assisted chemical vapour deposition of (CH_3_NH_3_) PbBr_3_, an inorganic-organic perovskite important in photovoltaics. Chem. Commun..

[69] Bhachu D.S., Scanlon D.O., Saban E.J., Bronstein H., Parkin I.P., Carmalt C.J., Palgrave R.G. (2015). Scalable route to CH_3_NH_3_PbI_3_ perovskite thin films by aerosol assisted chemical vapour deposition. J. Mater. Chem. A.

[70] Afzaal M., Yates H.M. (2017). Growth patterns and properties of aerosol-assisted chemical vapor deposition of CH_3_NH_3_PbI_3_ films in a single step. Surf. Coat. Technol..

[71] Liu Z.F., Luo P.F., Xia W., Zhou S.W., Cheng J.Q., Sun L., Xu C.X., Lu Y.W. (2016). Acceleration effect of chlorine in the gas-phase growth process of CH_3_NH_3_PbI_3_(Cl) films for efficient perovskite solar cells. J. Mater. Chem. C.

[72] Chen S., Briscoe J., Shi Y., Chen K., Wilson R.M., Dunn S., Binions R. (2015). A simple, low-cost CVD route to high-quality CH_3_NH_3_PbI_3_ perovskite thin films. CrystEngComm.

[73] Afzaal M., Salhi B., Al-Ahmed A., Yates H., Hakeem A. (2017). Surface-related properties of perovskite CH_3_NH_3_PbI_3_ thin films by aerosol-assisted chemical vapour deposition. J. Mater. Chem. C.

[74] Luo P.F., Zhou Y.G., Zhou S.W., Lu Y.W., Xu C.X., Sun L. (2018). Fast anion-exchange from CsPbI_3_ to CsPbBr_3_ via Br_2_-vapor-assisted deposition for air-stable all-inorganic perovskite solar cells. Chem. Eng. J..

[75] Peng Y., Jing G., Cui T. (2015). High crystalline quality perovskite thin films prepared by a novel hybrid evaporation/CVD technique. MRS Online Proc. Libr. Arch..

[76] Peng Y., Jing G., Cui T. (2015). A hybrid physical-chemical deposition process at ultra-low temperatures for high-performance perovskite solar cells. J. Mater. Chem. A.

[77] Ioakeimidis A., Christodoulou C., Lux-Steiner M., Fostiropoulos K. (2016). Effect of PbI_2_ deposition rate on two-step PVD/CVD all-vacuum prepared perovskite. J. Solid State Chem..

[78] Luo P.F., Zhou S.W., Liu Z.F., Xia W., Sun L., Cheng J.G., Xu C.X., Lu Y.W. (2016). A novel transformation route from PbS to CH_3_NH_3_PbI_3_ for fabricating curved and large-area perovskite films. Chem. Commun..

[79] Hossain M.I., Qarony W., Jovanov V., Tsang Y.H., Knipp D. (2018). Nanophotonic design of perovskite/silicon tandem solar cells. J. Mater. Chem. A.

[80] Jiang Y., Leyden M.R., Qiu L.B., Wang S.H., Ono L.K., Wu Z.F., Juarez-Perez E.J., Qi Y.B. (2018). Combination of hybrid CVD and cation exchange for upscaling Cs-substituted mixed cation perovskite solar cells with high efficiency and stability. Adv. Funct. Mater..

[81] Zhang Y.P., Liu J.Y., Wang Z.Y., Xue Y.Z., Ou Q.D., Polavarapu L., Zheng J.L., Qi X., Bao Q.L. (2016). Synthesis, properties, and optical applications of low-dimensional perovskites. Chem. Commun..

[82] Chen S., Shi G. (2017). Two-dimensional materials for halide perovskite-based optoelectronic devices. Adv. Mater..

[83] Dou L. (2017). Emerging two-dimensional halide perovskite nanomaterials. J. Mater. Chem. C.

[84] Fu P.F., Shan Q.S., Shang Y.Q., Song J.Z., Zeng H.B., Ning Z.J., Gong J.K. (2017). Perovskite nanocrystals: Synthesis, properties and applications. Sci. Bull..

[85] Yusoff A.R.B.M., Nazeeruddin M.K. (2018). Low-dimensional perovskites: From synthesis to stability in perovskite solar cells. Adv. Energy Mater..

[86] Hong K., Van Le Q., Kim S.Y., Jang H.W. (2018). Low-dimensional halide perovskites: Review and issues. J. Mater. Chem. C.

[87] Huang K., Liu J., Chang X., Fu L. (2019). Integrating properties modification in the synthesis of metal halide perovskites. Adv. Mater. Technol..

[88] Lee K.J., Turedi B., Sinatra L., Zhumekenov A.A., Maity P., Dursun I., Naphade R., Merdad N., Alsalloum A., Oh S. (2019). Perovskite-based artificial multiple quantum wells. Nano Lett..

[89] Lan C., Zhou Z., Wei R., Ho J.C. (2019). Two-dimensional perovskite materials: From synthesis to energy-related applications. Mater. Today Energy.

[90] Huo C.X., Cai B., Yuan Z., Ma B.W., Zeng H.B. (2017). Two-dimensional metal halide perovskites: Theory, synthesis, optoelectronics. Small Methods.

[91] Su L., Zhao Z.X., Li H.Y., Yuan J., Wang Z.L., Cao G.Z., Zhu G. (2015). High-performance organolead halide perovskite-based self-powered triboelectric photodetector. ACS Nano.

[92] Wang Y.P., Shi Y.F., Xin G.Q., Lan J., Shi J. (2015). Two-dimensional van der Waals epitaxy kinetics in a three-dimensional perovskite halide. Cryst. Growth Des..

[93] Li P.F., Shivananju B.N., Zhang Y.P., Li S.J., Bao Q.L. (2017). High performance photodetector based on 2D CH_3_NH_3_PbI_3_ perovskite nanosheets. J. Phys. D Appl. Phys..

[94] Qi X., Zhang Y.P., Ou Q.D., Ha S.T., Qiu C.W., Zhang H., Cheng Y.B., Xiong Q.H., Bao Q.L. (2018). Photonics and optoelectronics of 2D metal-halide perovskites. Small.

[95] Wen X.M., Chen W.J., Yang J.F., Ou Q.D., Yang T.S., Zhou C.H., Lin H., Wang Z.Y., Zhang Y.P., Conibeer G. (2018). Role of surface recombination in halide perovskite nanoplatelets. ACS Appl. Mater. Interfaces.

[96] Lan C., Dong R., Zhou Z., Shu L., Li D., Yip S., Ho J.C. (2017). Large-scale synthesis of freestanding layer-structured PbI_2_ and MAPbI_3_ nanosheets for high-performance photodetection. Adv. Mater..

[97] Chen J.N., Wang Y.G., Gan L., He Y.B., Li H.Q., Zhai T.Y. (2017). Generalized self-doping engineering towards ultrathin and large-sized two-dimensional homologous perovskites. Angew. Chem. Int. Ed..

[98] Kim Y.G., Kwon K.C., Van Le Q., Hong K., Jang H.W., Kim S.Y. (2016). Atomically thin two-dimensional materials as hole extraction layers in organolead halide perovskite photovoltaic cells. J. Power Sources.

[99] Bai F., Qi J.J., Li F., Fang Y.Y., Han W.P., Wu H.L., Zhang Y. (2018). A high-performance self-powered photodetector based on monolayer MoS_2_/Perovskite heterostructures. Adv. Mater. Interfaces.

[100] Zhou C.H., Ou Q.D., Chen W.J., Gan Z.X., Wang J., Bao Q.L., Wen X.M., Jia B.H. (2018). Illumination-induced halide segregation in gradient bandgap mixed-halide perovskite nanoplatelets. Adv. Opt. Mater..

[101] Luo Q., Ma H., Hou Q.Z., Li Y.X., Ren J., Dai X.Z., Yao Z.B., Zhou Y., Xiang L.C., Du H.Y. (2018). All-carbon-electrode-based endurable flexible perovskite solar cells. Adv. Funct. Mater..

[102] Meng X.Y., Zhou J.S., Hou J., Tao X., Cheung S.H., So S.K., Yang S.H. (2018). Versatility of carbon enables all carbon based perovskite solar cells to achieve high efficiency and high stability. Adv. Mater..

[103] Sung H., Ahn N., Jang M.S., Lee J.K., Yoon H., Park N.G., Choi M. (2016). Transparent conductive oxide-free graphene-based perovskite solar cells with over 17% efficiency. Adv. Energy Mater..

[104] Liu Z.K., You P., Xie C., Tang G.Q., Yan F. (2016). Ultrathin and flexible perovskite solar cells with graphene transparent electrodes. Nano Energy.

[105] You P., Liu Z., Tai Q.D., Liu S.H., Yan F. (2015). Efficient semitransparent perovskite solar cells with graphene electrodes. Adv. Mater..

[106] Kim S., Lee H.S., Kim J.M., Seo S.W., Kim J.H., Jang C.W., Choi S.H. (2018). Effect of layer number on flexible perovskite solar cells employing multiple layers of graphene as transparent conductive electrodes. J. Alloys Compd..

[107] Yoon J., Sung H., Lee G., Cho W., Ahn N., Jung H.S., Choi M. (2017). Superflexible, high-efficiency perovskite solar cells utilizing graphene electrodes: Towards future foldable power sources. Energy Environ. Sci..

[108] Heo J.H., Shin D.H., Jang M.H., Lee M.L., Kang M.G., Im S.H. (2017). Highly flexible, high-performance perovskite solar cells with adhesion promoted AuCl_3_-doped graphene electrodes. J. Mater. Chem..

[109] Heo J.H., Shin D.H., Song D.H., Kim D.H., Lee S.J., Im S.H. (2018). Super-flexible bis(trifluoromethanesulfonyl)-amide doped graphene transparent conductive electrodes for photo-stable perovskite solar cells. J. Mater. Chem..

[110] Lang F., Gluba M.A., Albrecht S., Rappich J., Korte L., Rech B., Nickel N.H. (2015). Perovskite solar cells with large-area CVD-graphene for tandem solar cells. J. Phys. Chem. Lett..

[111] Zhou J.X., Ren Z.W., Li S.H., Liang Z.C., Surya C., Shen H. (2018). Semi-transparent Cl-doped perovskite solar cells with graphene electrodes for tandem application. Mater. Lett..

[112] You P., Tang G., Yan F. (2019). Two-dimensional materials in perovskite solar cells. Mater. Today Energy.

[113] Hu X.H., Jiang H., Li J., Ma J.X., Yang D., Liu Z.K., Gao F., Liu S.Z. (2017). Air and thermally stable perovskite solar cells with CVD-graphene as the blocking layer. Nanoscale.

[114] Wang X.Y., Li Z., Xu W.J., Kulkarni S.A., Batabyal S.K., Zhang S., Cao A.Y., Wong L.H. (2015). TiO_2_ nanotube arrays based flexible perovskite solar cells with transparent carbon nanotube electrode. Nano Energy.

[115] Lee K.T., Guo L., Park H. (2016). Neutral-and multi-colored semitransparent perovskite solar cells. Molecules.

[116] Hodgkinson J.L., Yates H.M., Walter A., Sacchetto D., Moon S.J., Nicolay S. (2018). Roll to roll atmospheric pressure plasma enhanced CVD of titania as a step towards the realisation of large area perovskite solar cell technology. J. Mater. Chem. C.

[117] Bush K.A., Palmstrom A.F., Zhengshan J.Y., Boccard M., Cheacharoen R.R., Mailoa J.P., McMeekin D.P., Hoye R.L.Z., Bailie C.D., Leijtens T. (2017). 23.6%-efficient monolithic perovskite/silicon tandem solar cells with improved stability. Nat. Energy.

[118] Cheacharoen R.R., Rolston N., Harwood D., Bush K.A., Dauskardt R.H., McGehee M.D. (2018). Design and understanding of encapsulated perovskite solar cells to withstand temperature cycling. Energy Environ. Sci..

[119] Tong G.Q., Song Z.H., Li C.D., Zhao Y.L., Yu L.W., Xu J., Jiang Y., Sheng Y., Shi Y., Chen K.J. (2017). Cadmium-doped flexible perovskite solar cells with a low-cost and low-temperature-processed CdS electron transport layer. RSC Adv..

[120] Song Z.H., Tong G.Q., Li H., Li G.P., Ma S., Liu Q., Jiang Y. (2017). Three-dimensional architecture hybrid perovskite solar cells using CdS nanorod arrays as an electron transport layer. Nanotechnology.

[121] Ahn N., Jeon I., Yoon J., Kauppinen E.I., Matsuo Y., Maruyams S., Choi M. (2018). Carbon-sandwiched perovskite solar cell. J. Mater. Chem. A.

[122] Chen S.Q., Wang J.S., Zhang Z.X., Briscoe J., Warwick M.E.A., Li H.Y., Hu P. (2018). Aerosol assisted chemical vapour deposition of conformal ZnO compact layers for efficient electron transport in perovskite solar cells. Mater. Lett..

[123] Zhang Z.X., Chen S.Q., Li P.P., Li H.Y., Wu J.S., Hu P., Wang J.S. (2018). Aerosol-assisted chemical vapor deposition of ultra-thin CuO_x_ films as hole transport material for planar perovskite solar cells. Funct. Mater. Lett..

